# Supplementary material for: Hippocampal‐Prelimbic Coupling During Context‐Dependent Extinction Retrieval in Rats
Source: Hippocampus. 2025 Dec 23;36(1):e70058. doi: 10.1002/hipo.70058 (PMC12723576; doi:10.1002/hipo.70058)
Supplement: Supplementary file 1 — Figure S1: PLV permutation test applied across context exposure and pre‐CS periods. Each panel represents the experimental animal's null distribution, and the observed PLV values were transformed into Z‐scores relative to this distribution. Z‐values greater than 1.65 (dash lines) were considered statistically significant (one‐tailed; p < 0.05) Figure S2: Probability distribution function of locomotor activity bouts across experimental sessions. (A) Probability distribution of locomotor activity bouts (2‐s time windows) for context exposure, extinction pre‐CS, and extinction retrieval pre‐CS. (B) Cumulative probability function of locomotor activity bouts across each experimental session. (C) A two‐sample Kolmogorov–Smirnov test (KS test) was used to assess whether locomotor activity bouts came from the same distribution, comparing each experimental session. Figure S3: Pearson correlations between the relative magnitude envelope (6–8 Hz) and locomotor activity over the experimental sessions. (A) Over the pre‐CS period, data from all animals were extracted in sliding windows and averaged every 2 s. Theta‐band magnitude was computed by applying the Hilbert transform to the bandpass‐filtered signal (6–8 Hz), resulting in a complex analytic signal whose amplitude envelope corresponds to its magnitude. (B) Pearson correlations were computed individually for each experimental animal. The resulting coefficients were transformed using the Fisher Z‐transformation, which stabilizes the variance and renders the distribution approximately normal. The transformed values were then compared using analysis of variance (ANOVA), followed by pairwise comparisons with Tukey's post hoc test. p‐values are indicated in the figure. Figure S4: Phase synchrony between mPFC–PL and mPFC–IL. Delta phase vectors computed every 250 ms at 6–8 Hz during the pre‐CS period (gray lines), with the corresponding estimated mean phase values represented by arrows. The numbers above the polar plots indicate [file HIPO-36-0-s001.docx]

**Supplementary Figures**

**
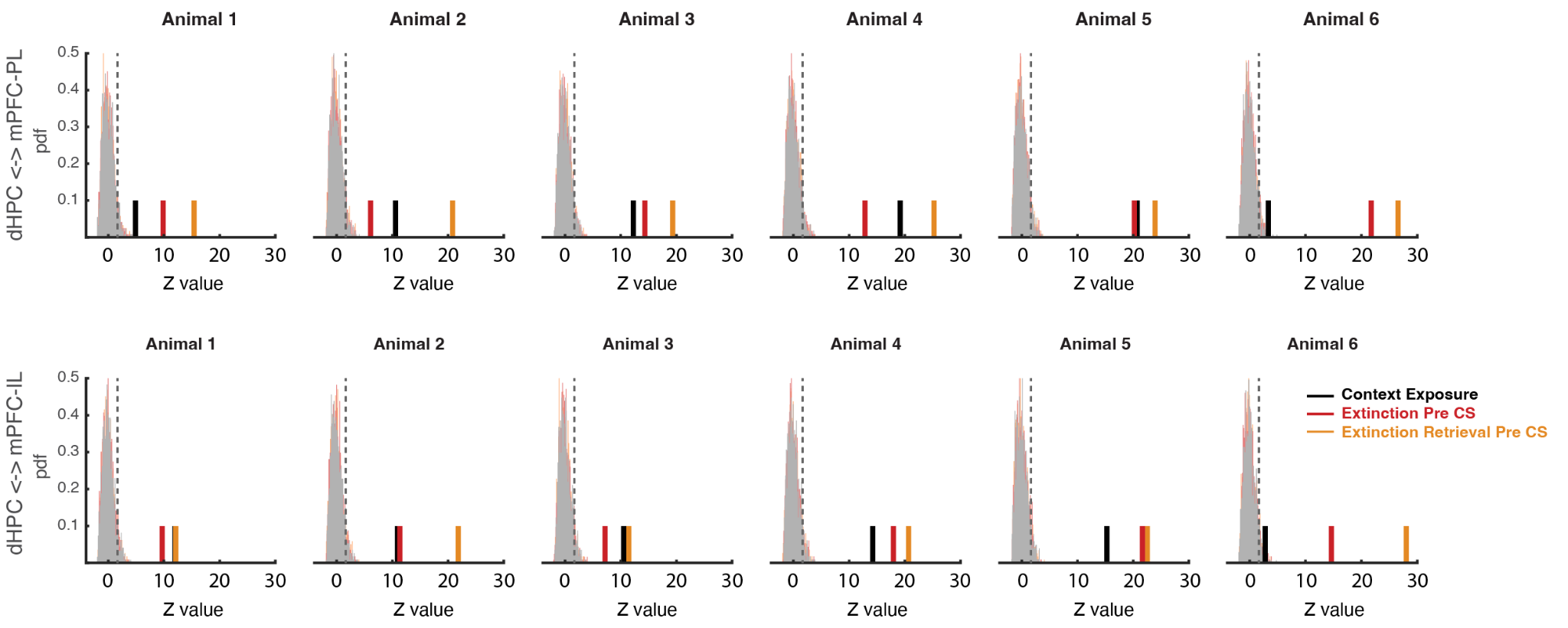
**

**Supplementary Figure 1: PLV permutation test applied across context exposure and pre-CS periods.** Each panel represents the experimental animal's null distribution, and the observed PLV values were transformed into *Z*-scores relative to this distribution. *Z*-values greater than 1.65 (dash lines) were considered statistically significant (one-tailed; *p* < 0.05)

**
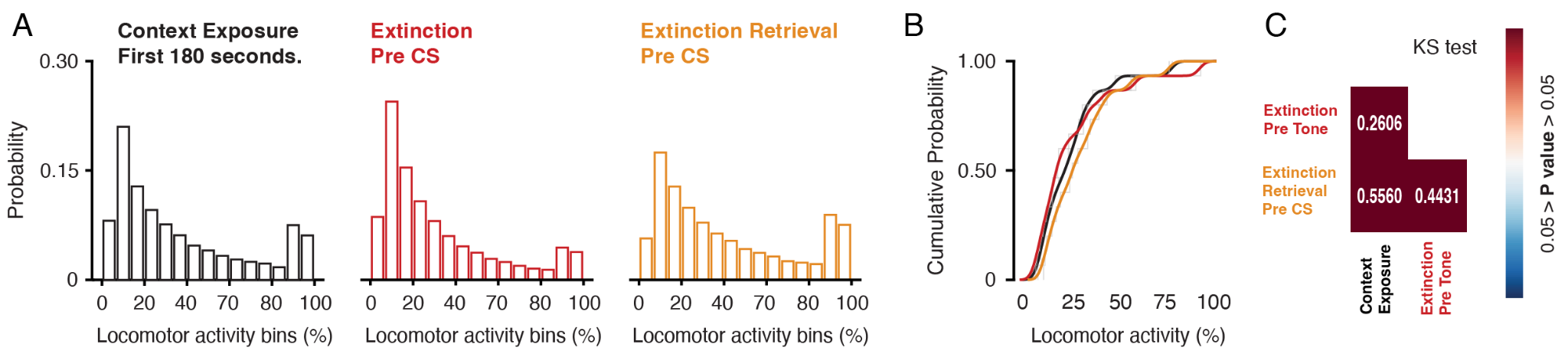
**

**Supplementary Figure 2: Probability distribution function of locomotor activity bouts across experimental sessions. A.** Probability distribution of locomotor activity bouts (2-sec time windows) for context exposure, extinction pre-CS, and extinction retrieval pre-CS. **B.** Cumulative probability function of locomotor activity bouts across each experimental session. **C.** A two-sample Kolmogorov–Smirnov test (KS test) was used to assess whether locomotor activity bouts came from the same distribution, comparing each experimental session.

**
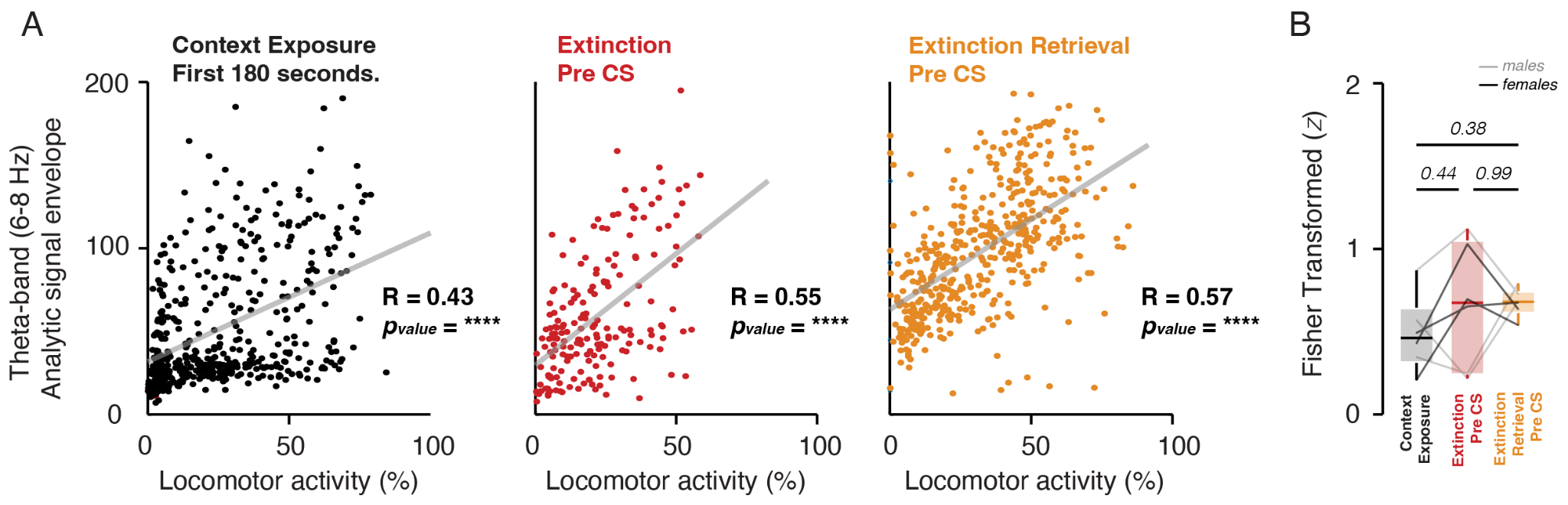
**

**Supplementary Figure 3: Pearson correlations between the relative magnitude envelope (6-8 Hz) and locomotor activity over the experimental sessions. A.** Over the pre-CS period, data from all animals were extracted in sliding windows and averaged every 2 seconds. Theta-band magnitude was computed by applying the Hilbert transform to the bandpass-filtered signal (6–8 Hz), resulting in a complex analytic signal whose amplitude envelope corresponds to its magnitude. **B.** Pearson correlations were computed individually for each experimental animal. The resulting coefficients were transformed using the Fisher *Z*-transformation, which stabilizes the variance and renders the distribution approximately normal. The transformed values were then compared using analysis of variance (ANOVA), followed by pairwise comparisons with Tukey’s post hoc test. *P-values* are indicated in the figure.

**
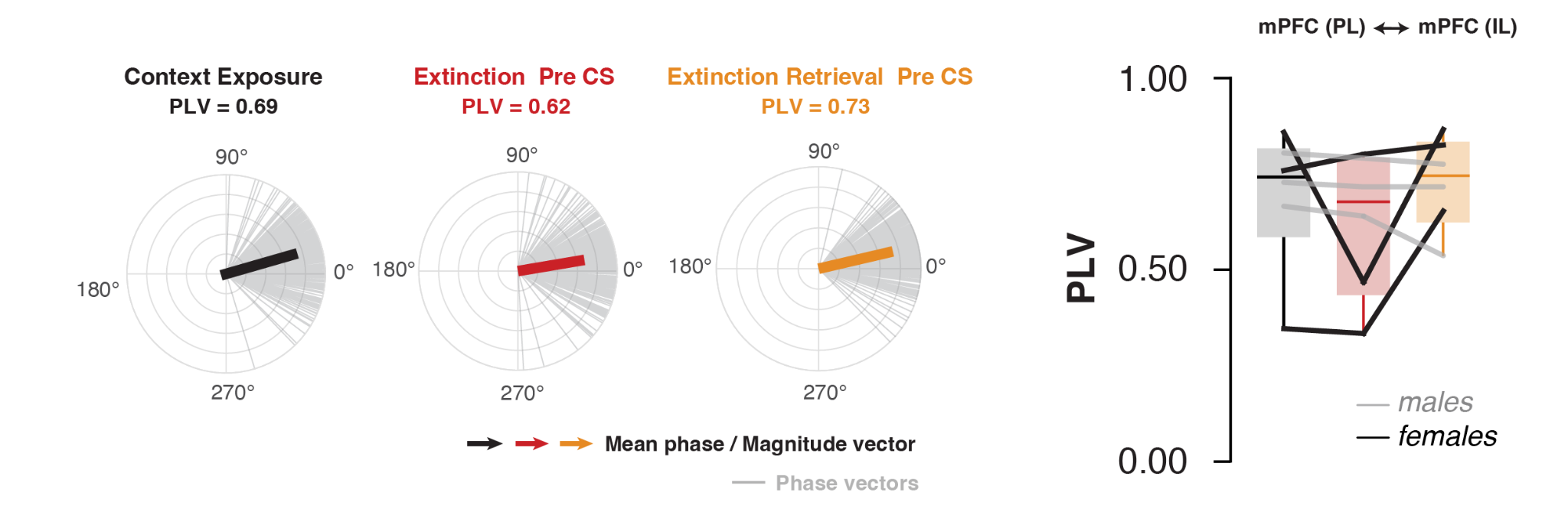
**

**Supplementary Figure 4: Phase synchrony between mPFC–PL and mPFC–IL.** Delta phase vectors computed every 250 ms at 6-8 Hz during the pre-CS period (gray lines), with the corresponding estimated mean phase values represented by arrows. The numbers above the polar plots indicate the mean phase-locking value (PLV) for each period. Analyses of synchrony did not reveal significant differences during contextual re-exposure across the experimental sessions.

*
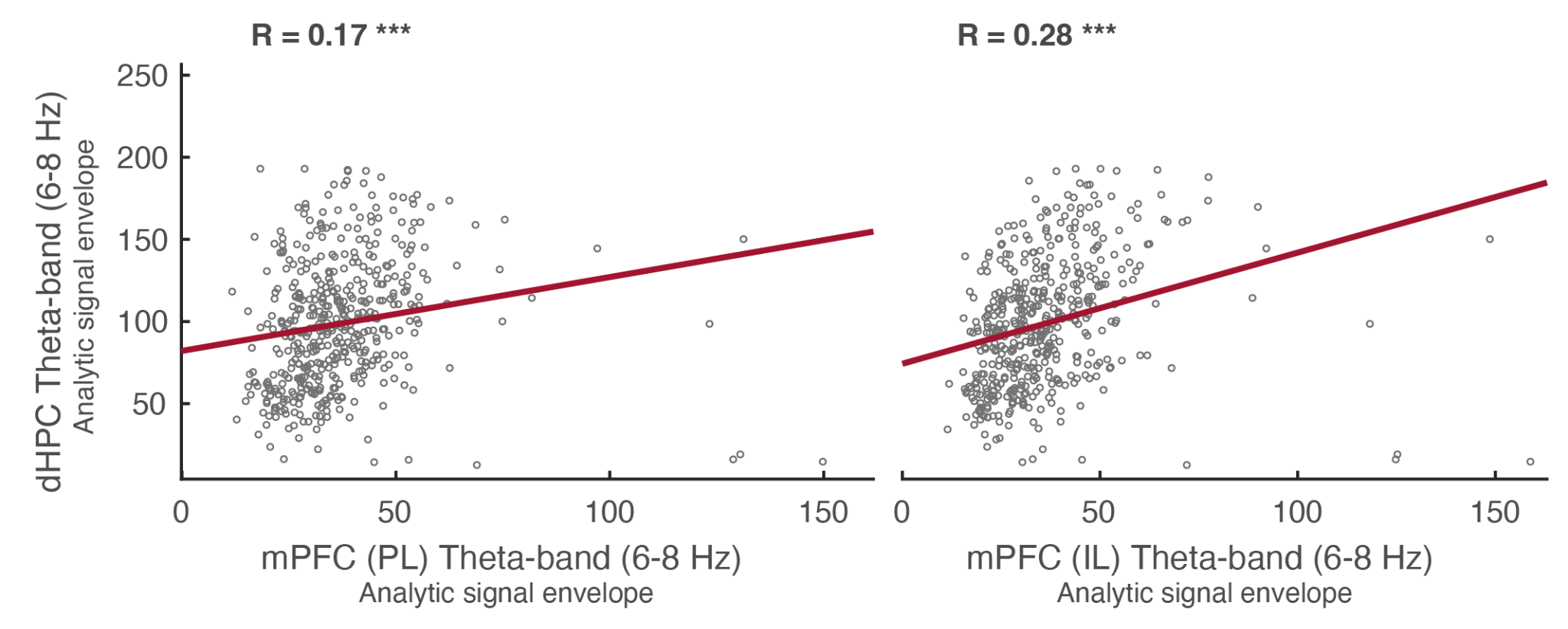
*

**Supplementary Figure 5: Correlations of the 6–8 Hz theta envelope between the dHPC and mPFC over extinction retrieval pre-CS:** Each panel shows the Pearson correlations (R) between the dHPC and the mPFC, computed using 2-sec time windows during the pre-CS period of extinction retrieval. The data were filtered between the 6–8 Hz and the amplitude envelope was extracted via the Hilbert transform.


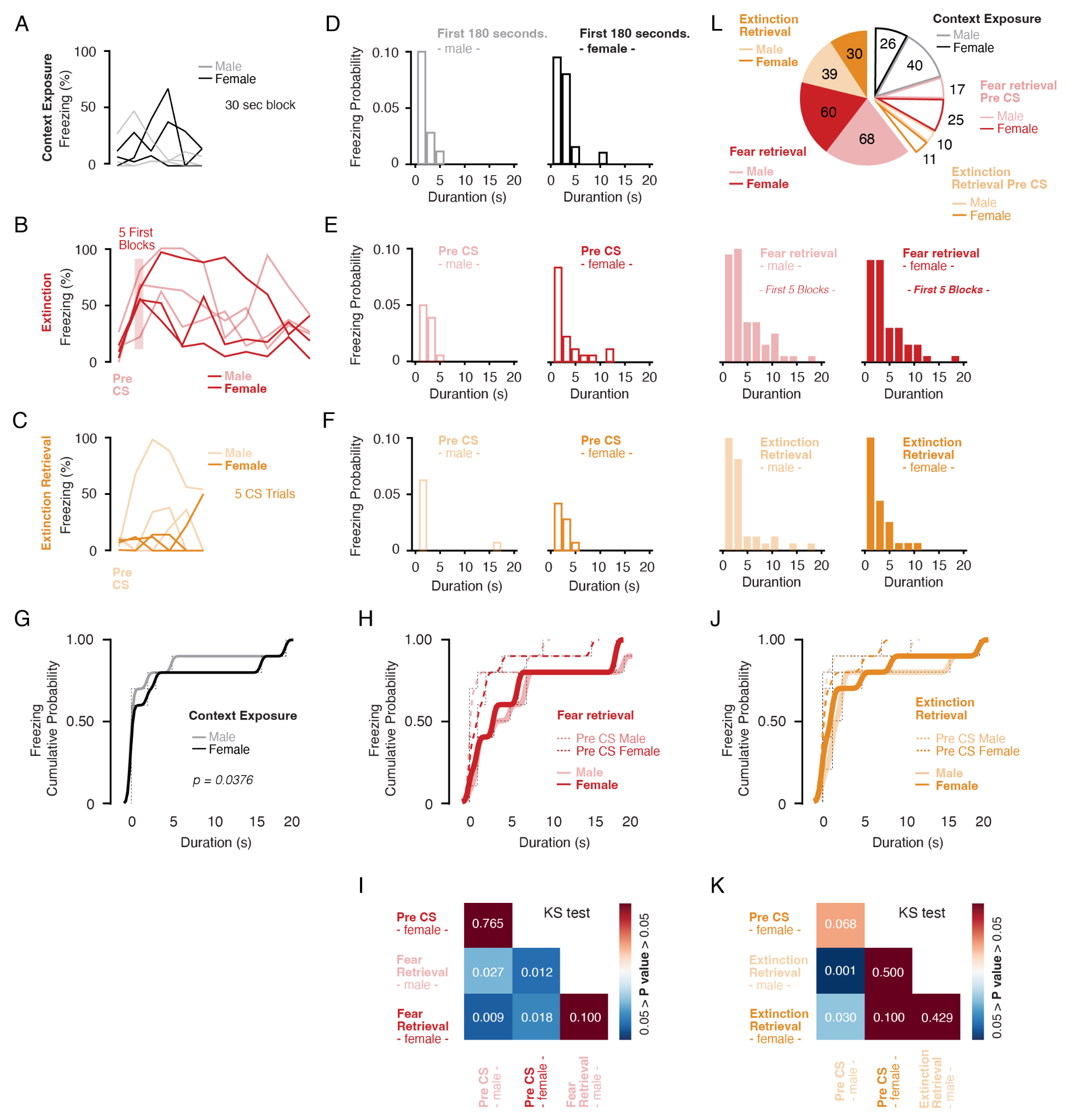


**Supplementary Figure 6:** **Distribution of conditioned freezing behavior across experimental sessions. A, B, C.** Percentage of freezing measured throughout each experimental session. The context exposure sample was divided into 30-sec time windows (totaling 180 sec). During extinction, freezing was measured in the pre-CS period followed by 45 CS trials, with the fear retrieval period considered over the first five trials. Extinction retrieval included the pre-CS period and five CS trials. Dark lines represent individual measurements from females, while lighter lines represent individual measurements from males. **D, E, F.** Probability distribution of freezing bout durations for context exposure, extinction pre-CS, fear retrieval, extinction retrieval pre-CS, and extinction retrieval CS presentations. **G, H, I.** Cumulative probability function of freezing bouts for males and females in each experimental session. **J, K.** A two-sample Kolmogorov–Smirnov test (KS test) was used to assess whether freezing bouts came from the same distribution, comparing each experimental phase for males and females. **L.** The pie chart represents the total number of freezing bouts per experimental session stratified by sex.
